# AluScan: a method for genome-wide scanning of sequence and structure variations in the human genome

**DOI:** 10.1186/1471-2164-12-564

**Published:** 2011-11-17

**Authors:** Lingling Mei, Xiaofan Ding, Shui-Ying Tsang, Frank W Pun, Siu-Kin Ng, Jianfeng Yang, Cunyou Zhao, Dezhi Li, Weiqing Wan, Chi-Hung Yu, Tze-Ching Tan, Wai-Sang Poon, Gilberto Ka-Kit Leung, Ho-Keung Ng, Liwei Zhang, Hong Xue

**Affiliations:** 1 Division of Life Science and Applied Genomics Centre, Hong Kong University of Science and Technology, 1 University Road, Clear Water Bay, Kowloon, Hong Kong, China; 2 Department of Neurosurgery, Beijing Tiantan Hospital, 6 Tiantan Xili, Dongcheng District, Capital Medical University, Beijing, 100050, China; 3 Chinese Cancer Genome Consortium, Beijing Genome Institute Shenzhen, 11 Beishan Industrial Zone, Yantian District, Shenzhen, 518083, China; 4 Department of Neurosurgery, Queen Elizabeth Hospital, 30 Gascoigne Road, Kowloon, Hong Kong, China; 5 Brain Cancer Genome Consortium - Hong Kong, Applied Genomics Center, Hong Kong University of Science and Technology, 1 University Road, Clear Water Bay, Kowloon, Hong Kong, China; 6 Division of Neurosurgery, Department of Surgery, Prince of Wales Hospital, Chinese University of Hong Kong, 30-32 Ngan Shing Street, Sha Tin, Hong Kong, China; 7 Division of Neurosurgery, Department of Surgery, Li Ka Shing Faculty of Medicine, University of Hong Kong, Queen Mary Hospital, 102 Pokfulam Road, Hong Kong, China; 8 Department of Anatomical and Cellular Pathology, Prince of Wales Hospital, Chinese University of Hong Kong, 30-32 Ngan Shing Street, Sha Tin, Hong Kong, China

## Abstract

**Background:**

To complement next-generation sequencing technologies, there is a pressing need for efficient pre-sequencing capture methods with reduced costs and DNA requirement. The Alu family of short interspersed nucleotide elements is the most abundant type of transposable elements in the human genome and a recognized source of genome instability. With over one million Alu elements distributed throughout the genome, they are well positioned to facilitate genome-wide sequence amplification and capture of regions likely to harbor genetic variation hotspots of biological relevance.

**Results:**

Here we report on the use of inter-Alu PCR with an enhanced range of amplicons in conjunction with next-generation sequencing to generate an Alu-anchored scan, or 'AluScan', of DNA sequences between Alu transposons, where Alu consensus sequence-based 'H-type' PCR primers that elongate outward from the head of an Alu element are combined with 'T-type' primers elongating from the poly-A containing tail to achieve huge amplicon range. To illustrate the method, glioma DNA was compared with white blood cell control DNA of the same patient by means of AluScan. The over 10 Mb sequences obtained, derived from more than 8,000 genes spread over all the chromosomes, revealed a highly reproducible capture of genomic sequences enriched in genic sequences and cancer candidate gene regions. Requiring only sub-micrograms of sample DNA, the power of AluScan as a discovery tool for genetic variations was demonstrated by the identification of 357 instances of loss of heterozygosity, 341 somatic indels, 274 somatic SNVs, and seven potential somatic SNV hotspots between control and glioma DNA.

**Conclusions:**

AluScan, implemented with just a small number of H-type and T-type inter-Alu PCR primers, provides an effective capture of a diversity of genome-wide sequences for analysis. The method, by enabling an examination of gene-enriched regions containing exons, introns, and intergenic sequences with modest capture and sequencing costs, computation workload and DNA sample requirement is particularly well suited for accelerating the discovery of somatic mutations, as well as analysis of disease-predisposing germline polymorphisms, by making possible the comparative genome-wide scanning of DNA sequences from large human cohorts.

## Background

Next-generation, massively-parallel sequencing technologies have transformed the landscape of genetics through their ability to produce giga-bases of sequence information in a single run. However, the sequencing cost, computation workload and amount of sample DNA required are still too high for large scale population analysis by means of whole-genome sequencing. There is clearly a need for pre-sequencing capture of subsets of the genome in order to reduce these requirements. Although the whole exome represents a valuable subset, its exclusion of introns, and the high cost and high DNA requirement for its analysis, remain major limitations. Other sequence subsets therefore clearly need to be explored.

Alu-transposons are a family of primate-specific short interspersed nucleotide elements (SINE) of ~ 300 bp derived from 7SL RNA [1]. Although Alu elements were once considered as 'junk DNA', their biological importance, in particular their influence on genome instability is being increasingly recognized [2,3]. They are abundant in gene-rich regions [4,5], exert a major impact on genomic architecture [6], and increase local recombination rates [7]. Previously we have found enhanced SNP frequencies in the vicinity of Alu-elements [8], more so among the youngest AluY elements than the intermediate-age AluS and the oldest AluJ. AluYs display also a higher rate of methylation, consistent with a stronger silencing pressure on these elements [9]. Genotypic variations surrounding a human lineage-specific AluY insertion in the *GABRB2 *gene encoding GABA_A _receptor β_2 _subunit have been found by us to constitute a joint focal point for positive evolutionary selection [10], hotspot recombinations [11] as well as association with schizophrenia and bipolar disorder [12,13]. Neighborhoods of Alu-transposons are therefore a highly significant sequence subset of the human genome in terms of evolutionary development and pathogenesis.

**Figure 1:**
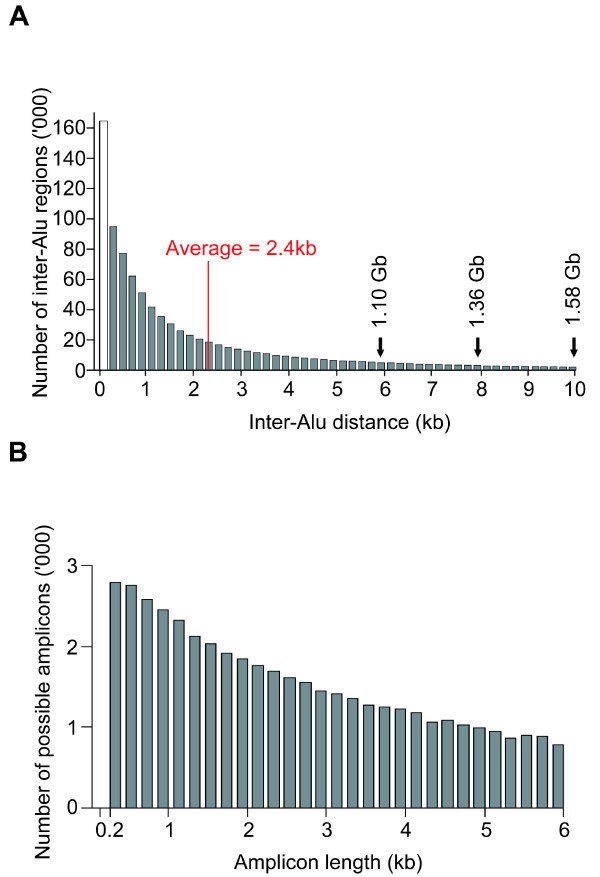
**Inter-Alu sequences in the human genome**. (A) Length distribution of inter-Alu distances between two adjacent Alu-transposons in the reference human genome. Heights of bars represent number of adjacent Alu pairs in the human genome at different inter-Alu distances. Subtraction of empty column at < 200 bp representing short inter-Alu sequences that were removed from analysis during library construction for next generation sequencing leaves the solid columns of inter-Alu sequences of varying lengths capturable from the genome. The total sum of the solid columns up to an inter-Alu distance of 6 kb, 8 kb or 10 kb equals 1.10 Gb, 1.36 Gb or 1.58 Gb, respectively. (B) Inter-Alu sequences of 0.2-6.0 kb in length that are in principle capturable by the three Alu-consensual primers AluY278T18, AluY66H21 and R12A/267. Such capturable amplicons amount to ~14 Mb if no mismatch is allowed between the consensual primers and template sequences, or ~106 Mb if one mismatch is allowed per primer. The graph shows the length distribution of the latter 106 Mb.

Inter-Alu PCR is a useful method for isolating human DNA in the presence of animal DNA [14], linkage mapping [15], creation of human specific probes and fingerprints [16], and detection of mutator phenotypes [17] or high frequency genetic alterations [18]. The general strategy of the method is to employ a single PCR primer based on the Alu consensus sequence to amplify the sequence between two Alu elements. With well over a million Alu-transposons in the human genome, the average distance between two Alus is only 2.4 kb (Figure 1A), which suggests that inter-Alu PCR with an enhanced amplicon range coupled to next-generation sequencing could yield a huge sequence subset of the human genome for analysis. Accordingly the objective of the present study is to examine the possibility of enhancing the amplicon range of inter-Alu PCR and combining it with next generation sequencing to scan for sequence and structure variations in the human genome.

## Results

**Figure 2:**
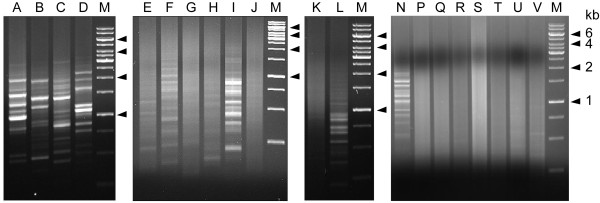
**Amplicon range of inter-Alu PCR**. The different lanes show gel electrophoretograms of amplicons obtained using different single primer or multiple primer sets: (A) AluJo56H16 alone; (B) AluJo232T16 alone; (C) AluSq56H16 alone; (D) AluSq263T16 alone; (E) AluJo56H16 and AluJo232T16; (F) AluJo56H16 and AluSq56H16; (G) AluJo56H16 and AluSq263T16; (H) AluJo232T16 and AluSq56H16; (I) AluJo232T16 and AluSq263T16; (J) AluSq56H16 and AluSq263T16; (K) AluJo56H16, AluSq56H16 and H-type L12A/8; (L) AluJo232T16, AluSq263T16 and T-type R12A/267, (N) AluY278T18, (P) AluY278T18 and AluY66H21, (Q)**-**(S) AluY278T18, AluY66H21 and R12A/267, (T) AluJo56H16, AluSq56H16 and AluSq263T16, (U) AluJo56H16, AluSq263T16 and AluJo232T16, (V) AluJo56H16, AluSq263T16 and L12A/8, and (M) M.W. markers. Notably, lanes E, G, H, J, P-V, where the inter-Alu PCR was performed using both H-type and T-type primers, gave rise to a largely smeared gel. Comparison of lanes N and P showed the conversion of a banded pattern obtained using a single T-type primer to a smeared one through the addition of an H-type primer. The much stronger staining intensity of lane S relative to lane R showed that the 0.30 μM concentrations of the same three primers in S compared to 0.10 μM in R resulted in increased amounts of amplicons. The primers AluJo56H16 (5'-GGCTCAAGCGATCCTC-3'), AluJo232T16 (5'-TATGATCGTGCCACTG-3'), AluSq56H16 (5'ACCTCAGGTGATCCAC-3'), and AluSq263T16 (5'-AACAAGAGCGAAACTC-3') were based on AluJo and AluSq consensus sequences [20]. H-type L12A/8 was an Alu consensus primer suggested earlier for inter-Alu PCR [15]. AluY278T18, AluY66H21 and T-type R12A/67 are described in Methods.

Individual Alu-transposons in the human genome are on the average only 15 - 20% divergent from each other, and PCR primers complementary to the Alu consensus sequence have been employed for inter-Alu PCR [14-18]. Likewise PCR primers based on consensus sequences in the AluJ, AluS and AluY subfamilies could also be devised. All Alu-based primers can be divided into 'H-type' where the primer extends outward from the head of the Alu, or 'T-type' where it extends outward from the poly-A containing tail. Previously, single general Alu consensus primers had given rise to agarose gel electrophoretograms displaying largely banded, banded plus smeared, or largely smeared patterns [14-18]. In the present study, varying combinations of Alu, AluJ, AluS and/or AluY consensus primers were found to yield widely different electrophoretogram patterns. The presence of a single H-type or T-type primer tended to yield a banded, non-smeared pattern suggestive of a limited amplicon range (lanes A-D, Figure 2). In lanes I and L respectively, even two or three T-type primers failed to give a non-banded pattern; lane K with three H-type primers gave a smeared pattern but lane F with two H-type primers gave only a banded pattern. In contrast, various primer combinations containing both H-type and T-type primers, allowing the amplification of intervening sequences between two Alu heads, between two Alu-tails as well as between one head and one tail, readily yielded a smeared gel indicating the presence of a wide diversity of amplicons of different sizes (lanes E, G, H, J, P-V). Therefore inclusion of both H-type and T-type primers provided the most reliable method for achieving huge amplicon range using no more than a small number of primers. The greater staining intensity of lane S compared to lane R further showed that the amounts of amplicons obtained from the same primer set could be increased by increasing the primer concentrations.

**Figure 3:**
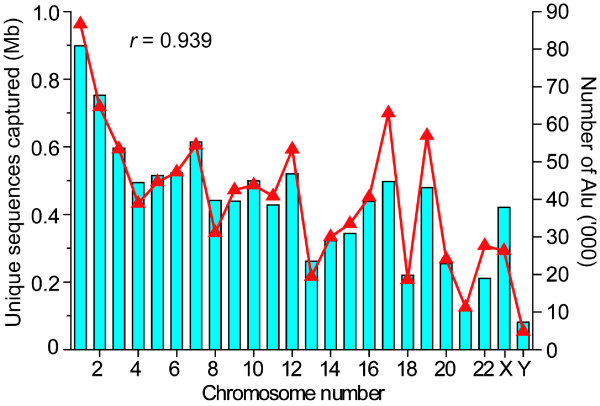
**Correlation between number of Alu elements and sequences captured**. Relationship between the number of Alu elements occurring on individual human chromosomes (blue bars) and the amount of AluScan-captured sequences of white blood cell DNA mapped to the different chromosomes (red triangles). Correlation coefficient was 0.939 between these quantities.

**Figure 4:**
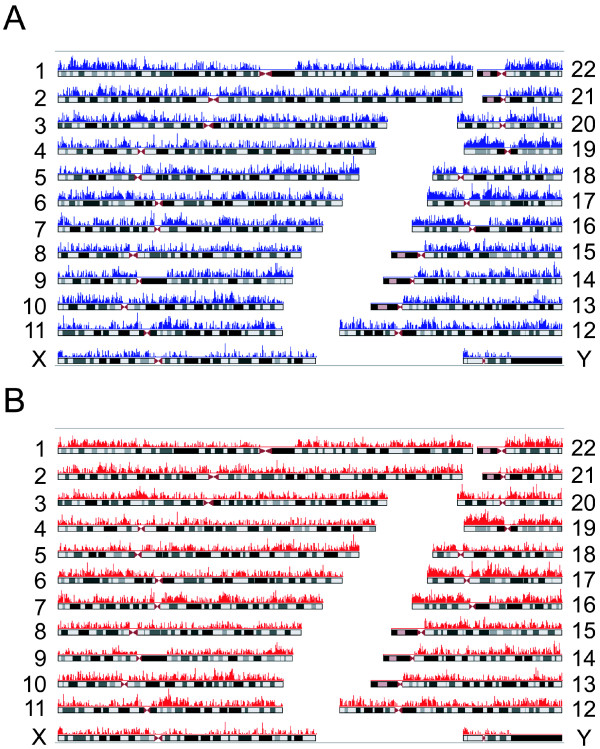
**Density distributions of captured sequences mapped on chromosomes**. The lengths of genomic sequences (blue or red peaks) mapped within 10-kb non-overlapping windows were plotted along chromosomes 1-22, X and Y using Human Genome Graphs (http://genome.ucsc.edu/cgi-bin/hgGenome) for (A) white blood cell DNA and (B) glioma DNA.

When AluScans were performed on paired control and cancer DNAs extracted from respectively the white blood cells and glioma tissue of a male Han Chinese patient using the three primers AluY278T18, AluY66H21 and R12A/267 described under Methods, smeared gels of amplicons up to ~ 6 kb in size were obtained (Figure 2, lane Q for control DNA). In each case the use of 90 ng sample DNA yielded sufficient amplicons for next-generation sequencing on the Illumina platform with a single flowcell lane and 75 bp paired-end reads. The sequencing output has been submitted to Sequence Read Archive (SRA) of NCBI. As indicated in Table 1, 837 Mb of the initial reads of control white blood cell DNA were mapped using the BWA program [19] to 58.9 Mb regions on the reference human genome (GRCh37.p2), including high quality mapping of 717 Mb reads to 10.6 Mb regions with minimum 10 times and average 67 times coverage. Of the latter 10.6 Mb, 95% were inter-Alu sequences, which compared favorably with the NimbleGen SeqCap Exome array for targeted exon capture with typically 71% mapped reads on target [20]; 53% were genic sequences including both exons and introns from 8,502 genes, representing an enrichment of genic sequences compared to the overall 40% gene content of the human genome; and 34% of the genes belonged to the list of cancer candidate genes in Gene Ranker: TCGA GBM 6000, exceeding the 26% of all human genes included in that list. The genomic regions mapped by the reads followed closely the number of Alu transposons located on the chromosomes (Figure 3). With glioma DNA, 984 Mb of the initial reads were mapped to 64.3 Mb genomic regions, including 11.8 Mb high quality regions with minimum 10 times and average 72 times coverage. The overlap between the high quality regions mapped by the control- and glioma-reads totalled 9.5 Mb, equal to 90% of the control-mapped regions; the correlation in read coverage between control and glioma reads was high, with r = 0.958; the density distributions of the control and glioma reads along different chromosomes (Figure 4) were also highly correlated, with r = 0.957. These results provided evidence that AluScan performed with the same set of primers enabled a reproducible genome-wide capture of DNA sequences that were enriched in both genic content and cancer candidate genes despite the many overlapping inter-Alu amplicons that might be amplified by a mixture of H- and T-type primers.

**Table 1:** Control and glioma sequence outputs from AluScan

	Control	Glioma
Total initial reads (bp)	896,661,780	1,065,234,324
Initial reads (bp) mapped to GRCh37.p2	836,745,196	983,798,813
Genomic regions mapped (bp) with coverage ≥ 1	58,876,070	64,297,421
Reads mapped (bp) with coverage ≥ 10	717,228,580	853,715,563
Genomic regions mapped with coverage ≥ 10:		
Total regions (bp)	10,638,683	11,846,868
Average depth (x)	67.4	72.1
Inter-Alu sequences mapped (bp)	10,149,445	11,275,690
Genic sequences mapped (bp)	5,644,023	6,274,433
Genic sequences mapped as % of total mapped regions	53.1%	53.0%
Excess in genic sequences^a^	13.1%	13.0%
Number of genes with mapped region	8,502	9,205
Number of cancer candidate genes^b ^with mapped region	2,921	3,096
Cancer candidate genes^b ^with mapped region as % of genes with mapped region	34.4%	33.6%
Excess in cancer candidate genes^c^	8.4%	7.6%

^a^Excess in % genic sequences with mapped region relative to the 40% genic sequences in the reference human genome.

^b^Cancer candidate genes refer to genes listed in Gene Ranker: TCGA GBM 6000.

^c^Excess in % cancer candidate genes with mapped region relative to the 26% of total human genes included in Gene Ranker: TCGA GBM 6000.

**Figure 5:**
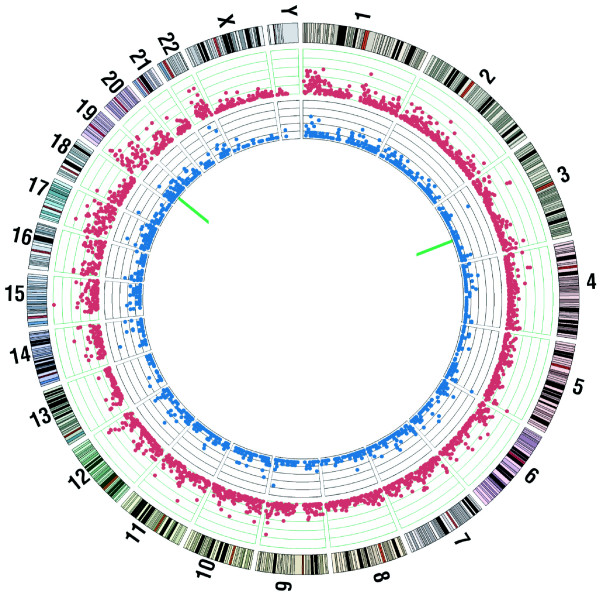
**Genetic variations identified in the white blood cell genome relative to the reference human genome**. From the outside, Tracks 1 and 2: numbering and cytobands of different chromosomes. Track 3: red dots showing number of SNVs per 1 Mb window (scale: 0-60, 1 div = 12). Track 4: blue dots showing number of indels of ≤ 30 bp per 1 Mb window (scale: 0-12, 1 div = 2.4). Inner circle: green bars showing positions of structural variations in the form of two large indels at chromosomes 3 and 19. The figure was drawn using the Circos program [29].

Figure 5 and Additional File 1 show the distributions of genetic variations occurring in the 10.6 Mb control genomic sequences relative to reference human genome among different chromosomes and types of genomic regions: there were 18,506 germline SNVs, 11,039 or 59.6% of which were novel SNVs absent from dbSNP132, 2,108 small (≤ 30 bp) indels and two larger indels, viz. a 75-bp deletion on chromosome 19 and a 767-bp deletion on chromosome 3. When 60 SNV-containing genic segments including 10 segments each containing a novel SNV were randomly chosen from the control AluScan output for Sanger sequencing, the accuracy of successful SNV verification was 81.6%.

**Figure 6:**
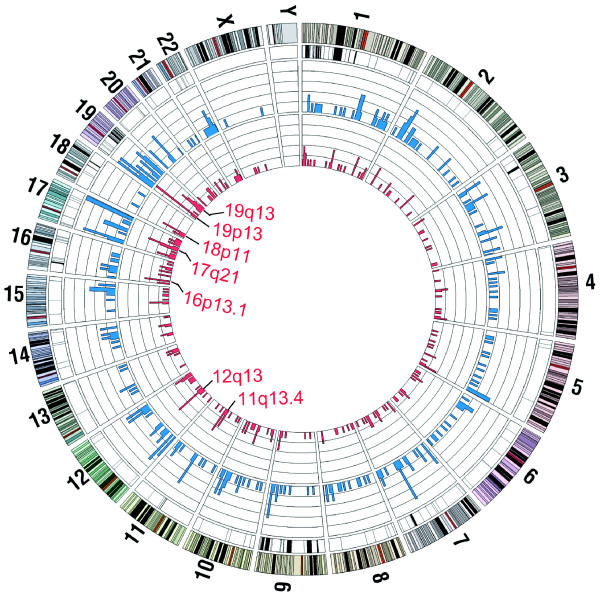
**Somatic mutations in glioma cells**. From the outsider, Tracks 1 and 2: numbering and cytobands of different chromosomes. Track 3: black bars showing the LOH-enriched loci in chromosomal regions, most prominently in 1p, 9p, 9q and 19q. Track 4: blue bars showing number of somatic indels of ≤ 30 bp per 5-Mb window (scale: 0-6). Track 5: red bars showing number of somatic SNVs per 5-Mb window (scale: 0-8). The cytogenetic locations of seven potential SNV hotspots displaying a density of somatic SNVs > 4, viz. greater than the sum of the Mean (= 1.5) plus two-times the standard deviation (= 1.9), per 5-Mb window are indicated as 11q13.4, 12q13, 16p13.1 etc.

Comparison of the mapped control and glioma sequences identified 274 somatic SNVs between them, 70.4% of which represented novel SNVs absent from dbSNP132. In the control and glioma SNVs relative to the reference human genome, as well as the somatic SNVs occurring between control and glioma, transitions were far more numerous than transversions (Additional File 2). There were 357 instances of loss of heterozygosity (LOH) and 341 somatic indels between control and glioma DNAs. The LOHs were unequally distributed among different chromosomes (Additional File 3). Of the four particularly LOH-enriched regions, viz. regions 1p, 9p, 9q and 19q indicated in Figure 6, notably 1p and 19q were known to contain glioma-associated deletions [21], which furnished valuable cross validation between AluScan and other genomic approaches.

Seven 5-Mb intervals in the glioma sequences displayed enhanced numbers of somatic SNVs, where the number of somatic SNVs > 4, indicating the potential presence of somatic SNV hotspots (Figure 6). Of these seven potential SNV hotspots, those in chromosomal regions 12q13, 17q21, 18p11, 19p13 and 19q13 harboured altogether 16 SNV-containing genes including *RAB5C *of the RAS oncogene family in 17q21 (Additional File 4). None of these 16 genes were included in OMIM as a known glioma-associated gene. These findings illustrated the usefulness of AluScan as a discovery tool.

## Discussion

Using only 90 ng sample DNA in each instance, the AluScans performed in the present study with one H-type and two T-type primers generated reads that covered a total of ~58-64 Mb, or ~1.9-2.1% of genomic sequences. This total was comparable in order of magnitude to the genomic sequences in principle capturable by the set of three H and T-type consensual Alu-based primers employed, which were estimated to be ~14 Mb for exact primer-template matches, or ~106 Mb allowing for one mismatched base-pair per primer (Figure 1B), but still far below the total of 1.10 Gb inter-Alu regions of ≤ 6 kb in length in the human genome (Figure 1A). Thus there could be ample room for widening the scope of AluScan-capturable sequences through the use of diverse combinations of H- and T-type primers. Primers specific for other transposable elements such as LINEs, LTRs, as well as other types of more specialized primers could also be utilized to tailor the AluScan capture to a given investigational goal. Moreover, by treating target DNA with bisulfite to modify unmethylated C-residues prior to AluScan, epigenomic changes in normal and diseased cells may also be monitored.

By combining the twin advantages of multitudinous amplification of inter-Alu sequences through the joint usage of H-type and T-type primers, and massively parallel next-generation sequencing, AluScan thus provides a new method for genome-wide investigation in addition to whole genome sequencing (WGS) and whole exome sequencing (WES). WGS is the standard in comprehensiveness, but incurs high operation cost, large computation workload and multi-microgram DNA requirement. WES provides integral insight into the entire exome, but leaves the intronic regions uncharacterized, besides incurring high capture cost and multi-microgram DNA requirement. AluScan permits an examination of gene-enriched segments of exons, introns and intergenic sequences requiring comparatively modest capture and sequencing costs, lighter computation workload and only sub-microgram DNA samples. These three methods complement one another, together making possible a comprehensive analysis of sequence and structure variations of the human genome.

## Conclusions

AluScan implemented with just a small number of PCR primers based on consensus Alu sequences provides a multiplex method for genome-wide sequence analysis. Through the inclusion of H and T type primers, the approach employs the abundance and wide distribution of Alu elements in the human genome as the basis for the effective capture of a huge number of DNA sequences in the vicinity of Alu elements. As demonstrated by the strong correlation between the captured white blood cell and glioma sequences, the same set of H and T-type primers has led to an extensively reproducible subset of genomic sequences in the two separate AluScans. As well, at least for this set of H and T-type primers, the captured sequences were enriched in genic and cancer-related DNA sequences.

The results in Figure 6 illustrate the utility of AluScan as a discovery tool. Comparison of the paired while blood cell-glioma DNAs of a single patient has led to the uncovering of 357 LOHs and 274 somatic SNVs, a majority of which likely arising in the glioma, and seven potential SNV hotspots located on six different chromosomes. Importantly, the modest technical cost and DNA sample size required for AluScan will render practicable a follow up with similarly paired AluScans for tens to hundreds of glioma patients in order to distinguish the somatic and germline driver mutations fundamental to the development of the disease from passenger mutations. A major application of AluScan will thus reside in its facilitation of large cohort studies for clinical and biological investigations of the human genome.

## Methods

### DNA samples

Paired blood and cancer samples were obtained with consent and institutional ethics approval from a male Chinese Han patient with anaplastic oligodendroglioma at Beijing Tiantan Hospital for the preparation of control DNA by phenol-chloroform extraction and cancer genomic DNA using the AllPrep kit (Qiagen).

### Inter-Alu PCR and next-generation sequencing

Fifteen parallel 25-μl PCR reaction mixtures each containing 2 μl Bioline 10× NH_4 _buffer (160 mM ammonium sulfate, 670 mM Tris-HCl, pH 8.8, 0.1% stabilizer), 3 mM MgCl_2_, 0.15 mM dNTP mix, 0.3 μM AluY278T18 primer, 0.18 μM AluY66H21 primer, 0.06 μM R12A/267 primer, 1 unit Bioline Taq polymerase, and 6 ng control or glioma DNA. PCR amplification for AluScan included DNA denaturation at 95°C for 5 min, followed by 35 cycles each of 30 s at 95°C, 30 s at 54°C, and 5 min at 71°C, and finally another 5 min at 71°C. Amplicons were purified with ethanol precipitation, and ≥ 3 μg purified products per sample were employed for Illumina GAII library construction and sequencing at Beijing Genomics Institute (Shenzhen, China). AluY278T18 (5'-GAGCGAGACTCCGTCTCA-3'), where 'AluY' represents the subfamily, '278' the first position on the AluY consensus sequence paired with the primer, 'T' a 'Tail-type' primer (vs. 'H' for 'H-type'), and '18' the length of the primer, and AluY66H21 (5'-TGGTCTCGATCTCCTGACCTC-3') were AluY consensus primers [22,23]. R12A/267 (T-type) was an Alu consensus primer employed earlier for inter-Alu PCR at an annealing temperature of 56°C [18].

### Agarose gel electrophoresis

PCR was performed basically as described in the preceding section, except that one PCR tube of 20 μl containing 100 ng control DNA was employed. The annealing temperatures were chosen to maximize in each instance the yield of amplicons: 60°C for lane A in Figure 2, 58°C for B-D, 56°C for H and L, 64°C for N, and 54°C for the other lanes. Primer concentration was 0.30 μM for the single-primer lanes A-D; 0.15 μM per primer for the two-primer lanes E-J; 0.10 μM per primer for the three-primer lanes K, L, R, T-V; 0.30 μM per primer for the triple-dosed lane S. The concentrations of primers AluY278T18, AluY66H21 and R12A/267 in lane Q were 0.375 μM, 0.225 μM, 0.075 μM respectively; lane P was same as lane Q with omission of R12A/267; and lane N was same as lane P with further omission of AluY66H21.

### Read mapping and variant analysis

Sequence reads were mapped to the GRCh37.p2 reference human genome using BWA (bwa-short algorithm version 0.5.9rc1) with default settings [19]. Initial mapping results were transferred into indexed and sorted BAM format using SAMtools version 0.1.12a [24], and further recalibrated and locally realigned using the Genome Analysis Toolkit (GATK version 1.0.4905) software [25]. Regions with read depths of < 10× were not analyzed further.

The UnifiedGenotyper module in GATK was used to produce the primary SNV calls, which were filtered using the parameter '-stand_call_conf 50.0' and the Variant Filtration module, ensuring a coverage depth > 10 x, mapping quality > 25.0 and strand bias < 0. SNVs in the vicinity of indels were removed by means of the IndelGenotyperV2 module. Further filtration was achieved using the criterion that homozygous reference loci have a non-reference read frequency of < 10%, heterozygous SNVs have a non-reference read frequency of ≥10% and <85%, and homozygous non-reference SNVs have a non-reference read frequency of ≥ 85%. Small indels were called using mpileup with '-ugf' and bcftools with '-bvcg' in SAMtools; and the calls were filtered using the script vcfutils.pl in SAMtools with default settings. Structural variants were identified initially using BreakDancer version 1.1 [26] and refined using Pindel version 0.20 [27]. Somatic SNVs were defined as heterozygous loci present in the tumor genome that corresponded to homozygous loci in the control genome, and LOH SNVs were defined as heterozygous loci present in the control genome that corresponded to homozygous loci in the tumor genome. Novel somatic SNVs were obtained by removing all LOHs and those SNVs already reported in dbSNP132. LOHs were identified by comparison between control and glioma reads using ExomeCNV version 1.23.0 [28].

## Authors' contributions

XD, SKN, FWP, JY, and CZ participated in data analysis. DL, GKKL, HKN, TCT, WSP, WW, HX, CHY and LZ developed the clinical approach and provided samples. HX, XD, LM, and SYT conceived the study, performed the experiments and helped draft the manuscript. All authors have read and approved the final manuscript.

## Supplementary Material

Additional File 1 **Distributions of SNVs and indels among different genomic regions**. Header: Control SNV = SNVs in control DNA relative to reference human genome GRCh37. Glioma SNV = SNVs in glioma DNA relative to reference human genome. Somatic SNV = SNVs between control and glioma DNAs. Control Indel = indels in control DNA relative to reference human genome. Glioma Indel = indels in glioma DNA relative to reference human genome. Somatic Indel = indels between control and glioma DNAs. LOH SNV = LOHs between control and glioma DNAs. Click here for file

Additional File 2 **Distribution of SNVs among different classes of nucleotidyl changes**. X-axis shows six different classes of possible nucleotidyl change in SNV, and Y-axis shows percentage of each class. Columns represent SNVs in control DNA relative to reference human genome (blue), in glioma DNA relative to reference human genome (red), and between the paired control and glioma DNAs (green). Click here for file

Additional File 3 **Distribution of LOHs on different chromosomes**. LOH regions are indicated in red, Non-LOH regions in yellow and unmapped regions in black, on horizon line in the diagram for each chromosome. Grey dots represent frequencies of non-reference alleles, found in either control or glioma SNVs that were not represented in the reference human genome. X axis shows position along each chromosome, and Y axis the non-reference allele frequency. Click here for file

Additional File 4 **Genic regions in potential SNV hotspots revealed by AluScans**. Header: Potential Hotspot = chromosomal location of each potential SNV hotspot shown on Figure 6. SNV position = positions of different genic SNVs in an indicated hotspot. Region = location of a genic SNV in a particular gene shown in 'Gene' column. Gene = name of gene in an indicated hotspot containing an SNV. Click here for file

## References

[B1] Ullu E Tschudi C Alu sequences are processed 7SL RNA genes Nature 1984 312 171 172 10.1038/312171a0 6209580

[B2] Konkel MK Batzer MA A mobile threat to genome stability: The impact of non-LTR retrotransposons upon the human genome Semin Cancer Biol 2010 20 211 221 10.1016/j.semcancer.2010.03.001 20307669 PMC2925057

[B3] Zhang Y Romanish MT Mager DL Distributions of transposable elements reveal hazardous zones in mammalian introns PLoS Comput Biol 2011 7 e1002046 10.1371/journal.pcbi.1002046 21573203 PMC3088655

[B4] Batzer MA Deininger PL Alu repeats and human genomic diversity Nat Rev Genet 2002 3 370 379 10.1038/nrg798 11988762

[B5] Lander ES Linton LM Birren B Nusbaum C Zody MC Baldwin J Devon K Dewar K Doyle M FitzHugh W Funke R Gage D Harris K Heaford A Howland J Kann L Lehoczky J LeVine R McEwan P McKernan K Meldrim J Mesirov JP Miranda C Morris W Naylor J Raymond C Rosetti M Santos R Sheridan A Sougnez C Initial sequencing and analysis of the human genome Nature 2001 409 860 921 10.1038/35057062 11237011

[B6] Deininger PL Moran JV Batzer MA Kazazian HH Jr Mobile elements and mammalian genome evolution Curr Opin Genet Dev 2003 13 651 658 10.1016/j.gde.2003.10.013 14638329

[B7] Witherspoon DJ Watkins WS Zhang Y Xing J Tolpinrud WL Hedges DJ Batzer MA Jorde LB Alu repeats increase local recombination rates BMC Genomics 2009 10 530 10.1186/1471-2164-10-530 19917129 PMC2785838

[B8] Ng SK Xue H Alu-associated enhancement of single nucleotide polymorphisms in the human genome Gene 2006 368 110 116 16380220 10.1016/j.gene.2005.10.034

[B9] Rodriguez J Vives L Jorda M Morales C Munoz M Vendrell E Peinado MA Genome-wide tracking of unmethylated DNA Alu repeats in normal and cancer cells Nucleic Acids Res 2008 36 770 784 18084025 10.1093/nar/gkm1105 PMC2241897

[B10] Lo WS Xu Z Yu Z Pun FW Ng SK Chen J Tong KL Zhao C Xu X Tsang SY Harano M Stober G Nimgaonkar VL Xue H Positive selection within the Schizophrenia-associated GABA_A _receptor β2 gene PLoS One 2007 2 e462 10.1371/journal.pone.0000462 17520021 PMC1866178

[B11] Ng SK Lo WS Pun FW Zhao C Yu Z Chen J Tong KL Xu Z Tsang SY Yang Q Yu W Nimgaonkar V Stober G Harano M Xue H A recombination hotspot in a schizophrenia-associated region of *GABRB2* PLoS One 2010 5 e9547 10.1371/journal.pone.0009547 20221451 PMC2833194

[B12] Lo WS Lau CF Xuan Z Chan CF Feng GY He L Cao ZC Liu H Luan QM Xue H Association of SNPs and haplotypes in GABA_A _receptor β2 gene with schizophrenia Mol Psychiatry 2004 9 603 608 10.1038/sj.mp.4001461 14699426

[B13] Zhao C Xu Z Wang F Chen J Ng SK Wong PW Yu Z Pun FW Ren L Lo WS Tsang SY Xue H Alternative-splicing in the exon-10 region of GABA_A _receptor β2 subunit gene: relationships between novel isoforms and psychotic disorders PLoS One 2009 4 e6977 10.1371/journal.pone.0006977 19763268 PMC2741204

[B14] Nelson DL Ledbetter SA Corbo L Victoria MF Ramirez-Solis R Webster TD Ledbetter DH Caskey CT Alu polymerase chain reaction: a method for rapid isolation of human-specific sequences from complex DNA sources Proc Natl Acad Sci USA 1989 86 6686 6690 10.1073/pnas.86.17.6686 2771952 PMC297910

[B15] Zietkiewicz E Labuda M Sinnett D Glorieux FH Labuda D Linkage mapping by simultaneous screening of multiple polymorphic loci using Alu oligonucleotide-directed PCR Proc Natl Acad Sci USA 1992 89 8448 8451 10.1073/pnas.89.18.8448 1528850 PMC49937

[B16] Kass DH Batzer MA Inter-Alu polymerase chain reaction: advancements and applications Anal Biochem 1995 228 185 193 10.1006/abio.1995.1338 8572294

[B17] Krajinovic M Richer C Labuda D Sinnett D Detection of a mutator phenotype in cancer cells by inter-Alu polymerase chain reaction Cancer Res 1996 56 2733 2737 8665504

[B18] Srivastava T Seth A Datta K Chosdol K Chattopadhyay P Sinha S Inter-alu PCR detects high frequency of genetic alterations in glioma cells exposed to sub-lethal cisplatin Int J Cancer 2005 117 683 689 10.1002/ijc.21057 15912534

[B19] Li H Durbin R Fast and accurate short read alignment with Burrows-Wheeler transform Bioinformatics 2009 25 1754 1760 10.1093/bioinformatics/btp324 19451168 PMC2705234

[B20] Kim DW Nam SH Kim RN Choi SH Park HS Whole human exome capture for high-throughput sequencing Genome 2010 53 568 574 10.1139/G10-025 20616878

[B21] Durand KS Guillaudeau A Weinbreck N DeArmas R Robert S Chaunavel A Pommepuy I Bourthoumieu S Caire F Sturtzand FG Labrousse FJ 1p19q LOH patterns and expression of p53 and Olig2 in gliomas: relation with histological types and prognosis Mol Pathol 2010 23 619 628 10.1038/modpathol.2009.185 20081802

[B22] Park ES Huh JW Kim TH Kwak KD Kim W Kim HS Analysis of newly identified low copy AluYj subfamily Genes Genet Syst 2005 80 415 422 10.1266/ggs.80.415 16501310

[B23] Price AL Eskin E Pevzner PA Whole-genome analysis of Alu repeat elements reveals complex evolutionary history Genome Res 2004 14 2245 2252 10.1101/gr.2693004 15520288 PMC525682

[B24] Li H Handsaker B Wysoker A Fennell T Ruan J Homer N Marth G Abecasis G Durbin R The Sequence Alignment/Map format and SAMtools Bioinformatics 2009 25 2078 2079 10.1093/bioinformatics/btp352 19505943 PMC2723002

[B25] McKenna A Hanna M Banks E Sivachenko A Cibulskis K Kernytsky A Garimella K Altshuler D Gabriel S Daly M DePristo MA The Genome Analysis Toolkit: a MapReduce framework for analyzing next-generation DNA sequencing data Genome Res 2010 20 1297 1303 10.1101/gr.107524.110 20644199 PMC2928508

[B26] Chen K Wallis JW McLellan MD Larson DE Kalicki JM Pohl CS McGrath SD Wendl MC Zhang Q Locke DP Shi X Fulton RS Ley TJ Wilson RK Ding L Mardis ER BreakDancer: an algorithm for high-resolution mapping of genomic structural variation Nat Methods 2009 6 677 681 10.1038/nmeth.1363 19668202 PMC3661775

[B27] Ye K Schulz MH Long Q Apweiler R Ning Z Pindel: a pattern growth approach to detect break points of large deletions and medium sized insertions from paired-end short reads Bioinformatics 2009 25 2865 2871 10.1093/bioinformatics/btp394 19561018 PMC2781750

[B28] Sathirapongsasuti JF Lee H Horst BA Brunner G Cochran AJ Binder S Quackenbush J Nelson SF Exome sequencing-based copy-number variation and loss of heterozygosity detection: ExomeCNV Bioinformatics 2011 27 2648 2654 10.1093/bioinformatics/btr462 21828086 PMC3179661

[B29] Krzywinski M Schein J Birol I Connors J Gascoyne R Horsman D Jones SJ Marra MA Circos: an information aesthetic for comparative genomics Genome Res 2009 19 1639 1645 10.1101/gr.092759.109 19541911 PMC2752132

