# Supplementary material for: AluScan: a method for genome-wide scanning of sequence and structure variations in the human genome
Source: BMC Genomics. 2011 Nov 17;12:564. doi: 10.1186/1471-2164-12-564 (PMC3228862; doi:10.1186/1471-2164-12-564)
Supplement: Additional File 1 — Distributions of SNVs and indels among different genomic regions. Header: Control SNV = SNVs in control DNA relative to reference human genome GRCh37. Glioma SNV = SNVs in glioma DNA relative to reference human genome. Somatic SNV = SNVs between control and glioma DNAs. Control Indel = indels in control DNA relative to reference human genome. Glioma Indel = indels in glioma DNA relative to reference human genome. Somatic Indel = indels between control and glioma DNAs. LOH SNV = LOHs between control and glioma DNAs. [file 1471-2164-12-564-S1.PDF]

Additional File 1-Distributions of SNVs and indels among different genomic regions

Regions	Control SNV	Glioma SNV	Somatic SNV	Control Indel	Glioma Indel	Somatic Indel	LOH SNV
***Alu Neighborhoods*:**							
**Inside Alu**	12,590	14,156	199	1,296	1,373	228	193
**0-10bp from Alu**	294	356	15	135	148	29	9
**10-100 bp from Alu**	2,334	2,570	35	296	315	40	84
**100-200 bp from Alu**	1,378	1,412	16	187	188	25	40
**200-1k bp from Alu**	1,832	1,826	8	187	182	19	29
**1-6k bp from Alu**	64	62	1	5	3	0	1
***Genic region*:**	9,847	10,945	149	1,060	1,121	185	180
**Exon**	176	200	3	11	22	2	2
**Intron**	9,760	10,744	147	1,053	1,108	184	179
**Splice sites**	2	2	0	0	1	0	0
**5’UTR**	19	23	0	1	1	0	0
**3’UTR**	80	97	1	7	15	1	1
Intergenic region	8,659	9,449	125	1,048	1,089	156	177
CpG dinucleotides	1,978	2,221	27	132	136	20	34
PolyA tail	3	3	0	2	3	0	0
Total	**18,506**	**20,394**	**274**	**2,108**	**2,210**	**341**	**357**
