# Supplementary figures and images for: AluScan: a method for genome-wide scanning of sequence and structure variations in the human genome
Source: BMC Genomics. 2011 Nov 17;12:564. doi: 10.1186/1471-2164-12-564 (PMC3228862; doi:10.1186/1471-2164-12-564)

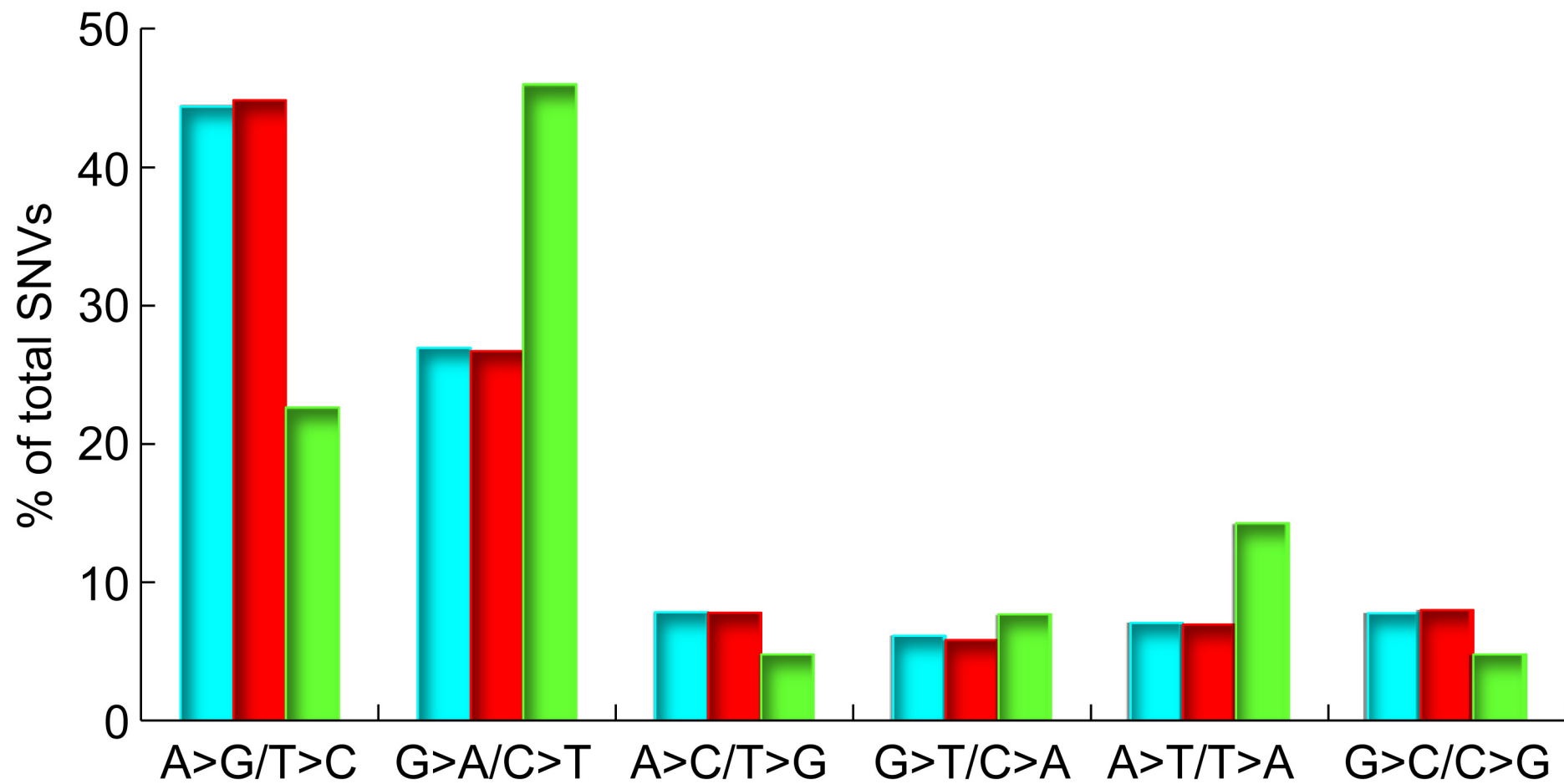

Supplement: Additional File 2 — Distribution of SNVs among different classes of nucleotidyl changes. X-axis shows six different classes of possible nucleotidyl change in SNV, and Y-axis shows percentage of each class. Columns represent SNVs in control DNA relative to reference human genome (blue), in glioma DNA relative to reference human genome (red), and between the paired control and glioma DNAs (green). [file 1471-2164-12-564-S2.PDF]

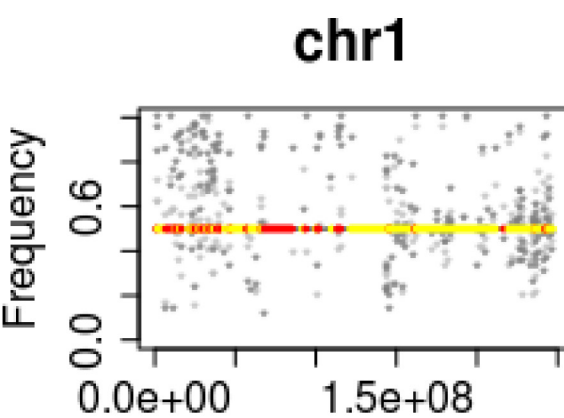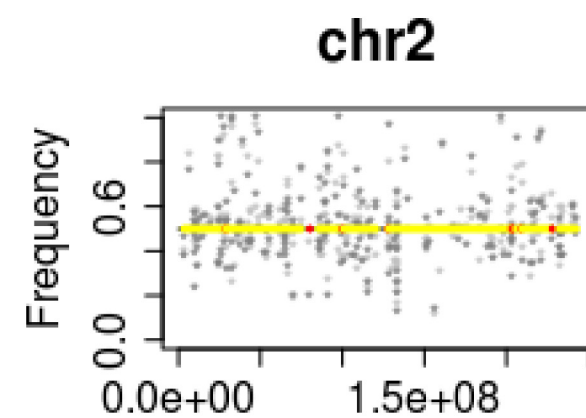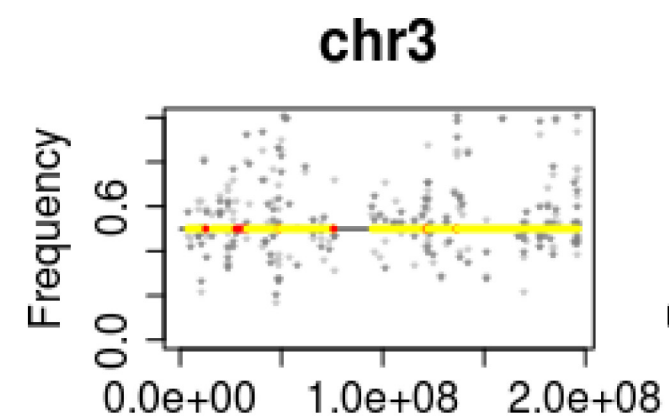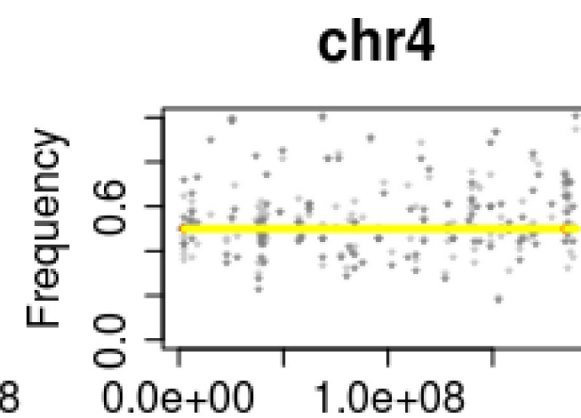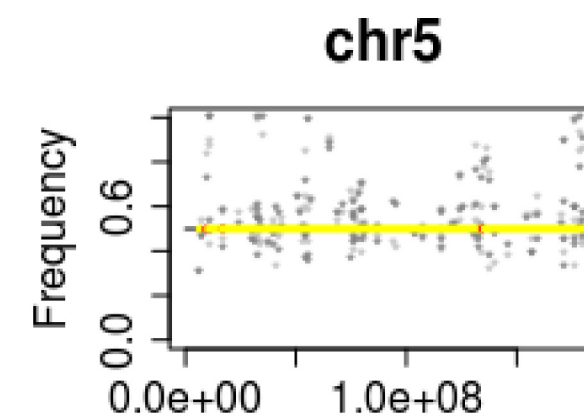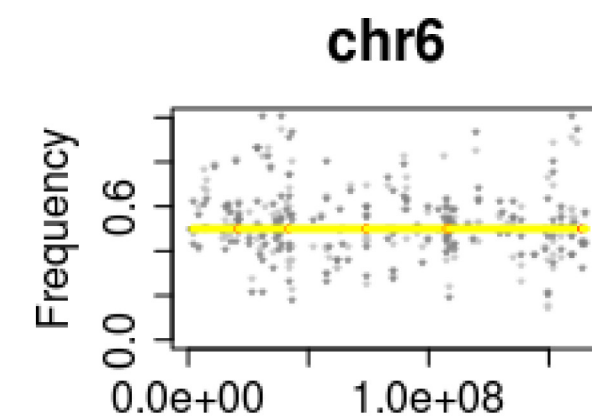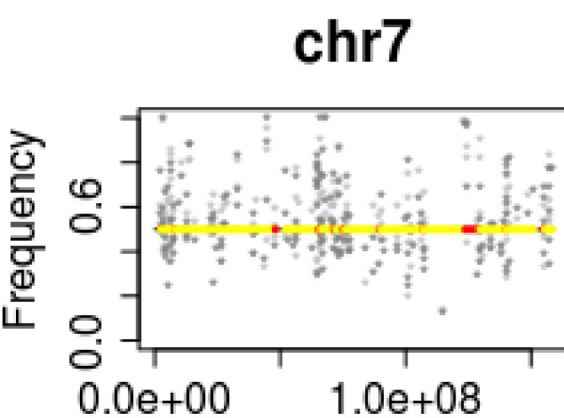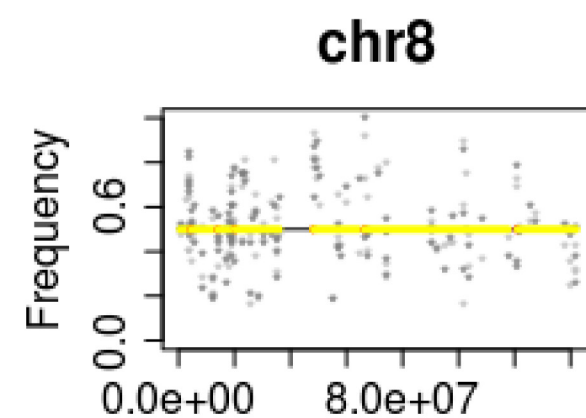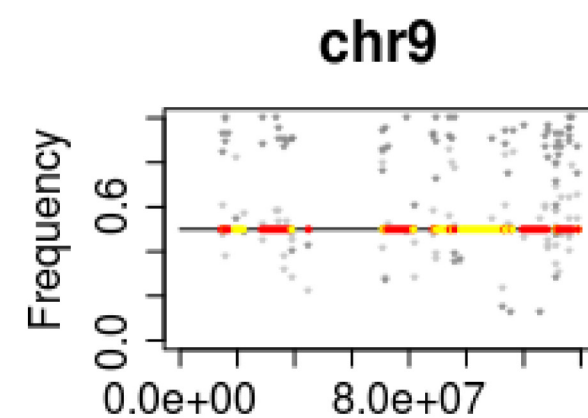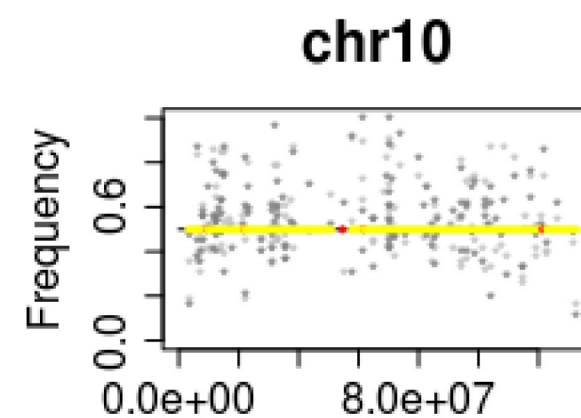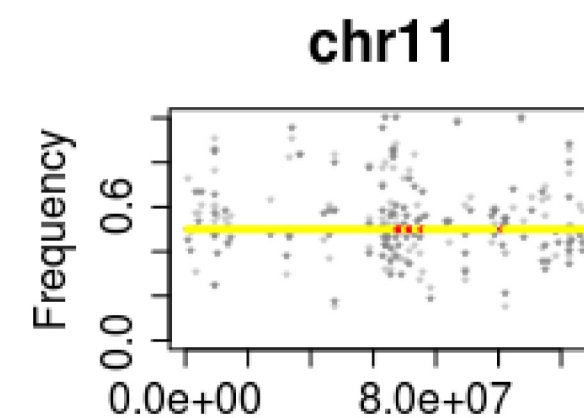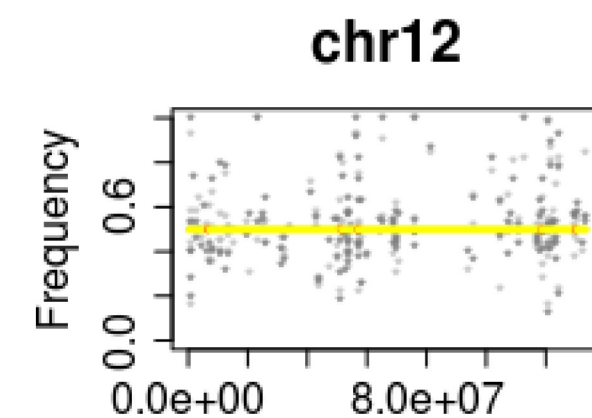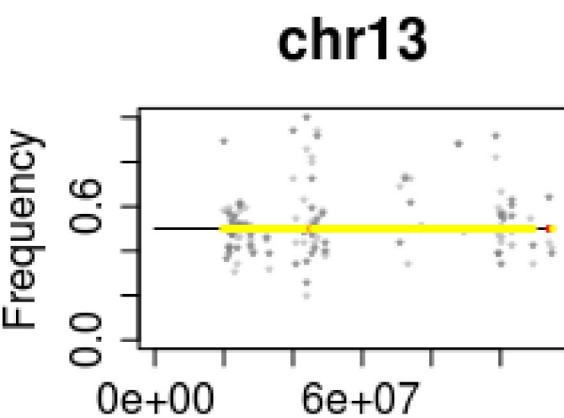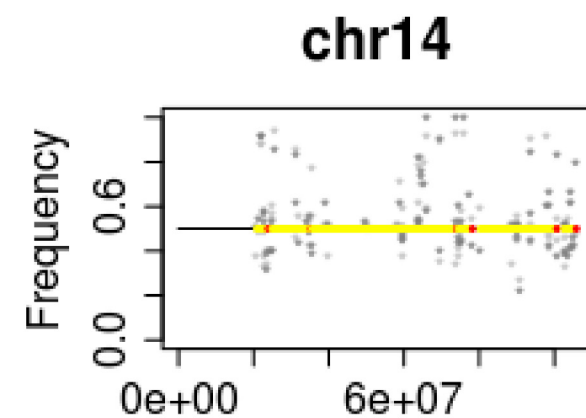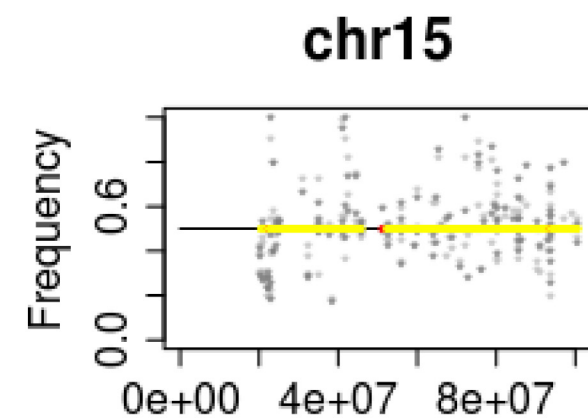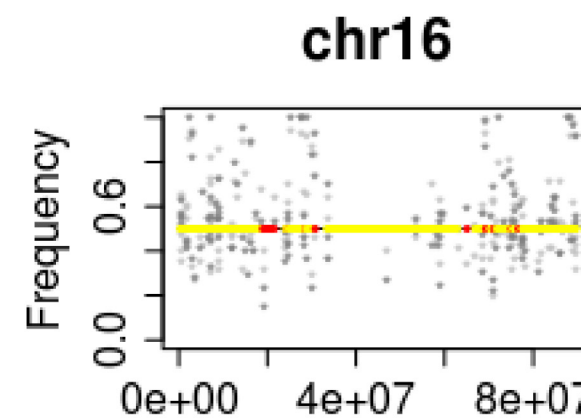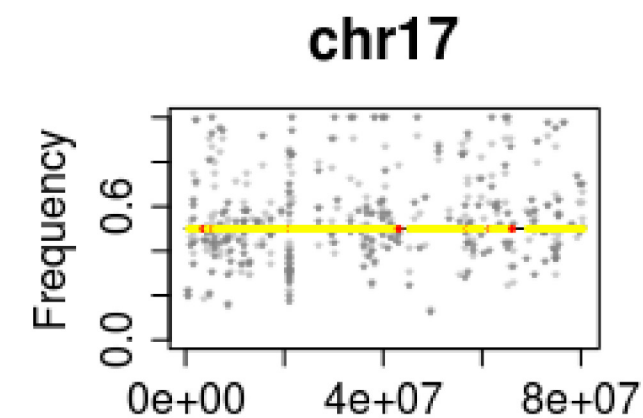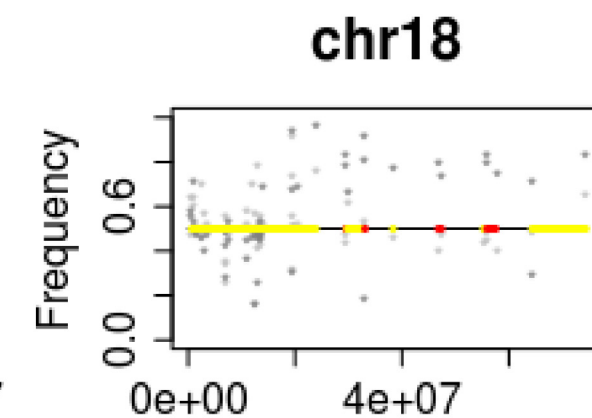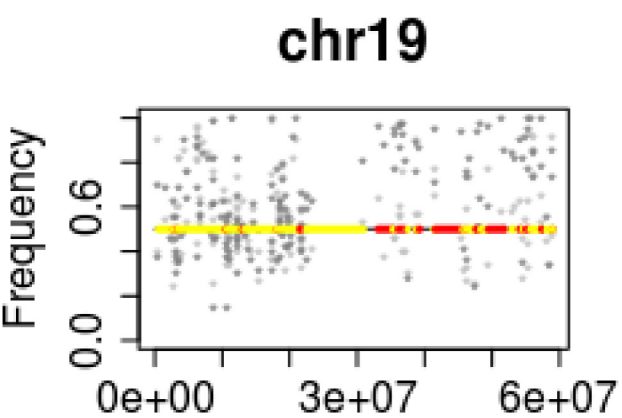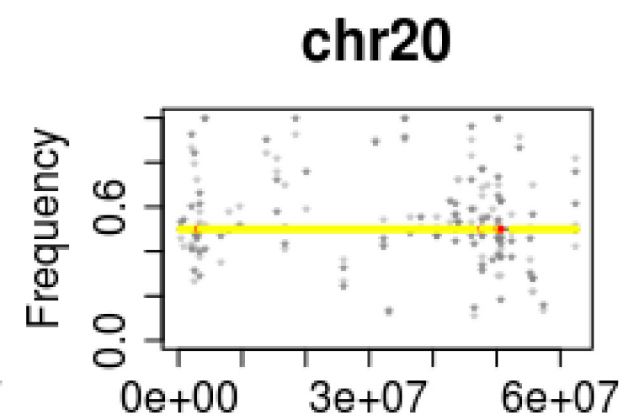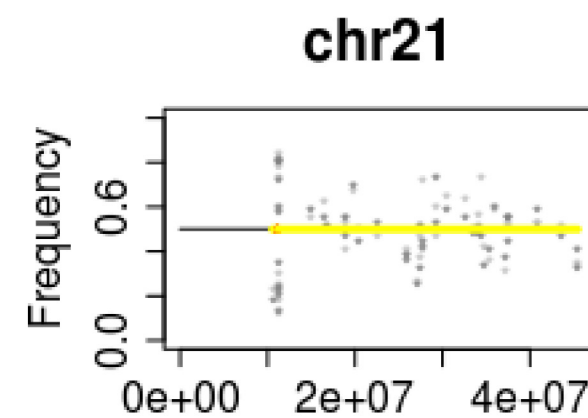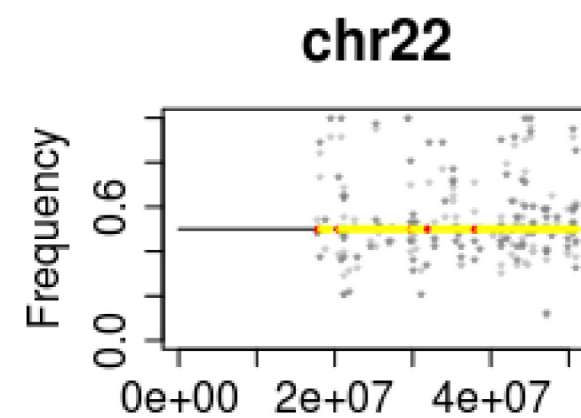

Supplement: Additional File 3 — Distribution of LOHs on different chromosomes. LOH regions are indicated in red, Non-LOH regions in yellow and unmapped regions in black, on horizon line in the diagram for each chromosome. Grey dots represent frequencies of non-reference alleles, found in either control or glioma SNVs that were not represented in the reference human genome. X axis shows position along each chromosome, and Y axis the non-reference allele frequency. [file 1471-2164-12-564-S3.PDF]
