# Supplementary material for: AluScan: a method for genome-wide scanning of sequence and structure variations in the human genome
Source: BMC Genomics. 2011 Nov 17;12:564. doi: 10.1186/1471-2164-12-564 (PMC3228862; doi:10.1186/1471-2164-12-564)
Supplement: Additional File 4 — Genic regions in potential SNV hotspots revealed by AluScans. Header: Potential Hotspot = chromosomal location of each potential SNV hotspot shown on Figure 6. SNV position = positions of different genic SNVs in an indicated hotspot. Region = location of a genic SNV in a particular gene shown in 'Gene' column. Gene = name of gene in an indicated hotspot containing an SNV. [file 1471-2164-12-564-S4.PDF]

Additional File 4-Genic regions in potential SNV hotspots revealed by AluScans

Potential Hotspot	SNV position	Region	Gene
12q13	53920401(G/A)	Intron 8	*ATF7* (activating transcription factor 7)
	56987232(C/G)	3'UTR	*RBMS2* (RNA binding motif, single stranded interacting protein 2)
	50866622(A/G)	Intron 13	*LARP4* (La ribonucleoprotein domain family, member 4)
17q21	38433040(C/T)	Intron 6	*WIPF2* (WAS/WASL interacting protein family, member 2)
	40303451(T/A)	Intron 1	*RAB5C* (RAB5C, member RAS oncogene family)
	43140569(T/A)	Intron 1	*NMT1* (N-myristoyltransferase 1)
18p11	12654818(G/A)	Intron 1	*SPIRE1* (spire homolog 1 Drosophila)
19p13	12162103(A/G)	Intron 2	*ZNF878* (zinc finger protein 878)
	13083661(G/T)	Intron 1	*DAND5* (DAN domain family, member 5)
19q13	39515175(C/T)	3'UTR	*FBXO27* (F-box protein 27)
	40976768(C/G)	Intron 1	*SPTBN4* (spectrin, beta, non-erythrocytic 4)
	41788391(C/T)	Intron 7	*HNRNPUL1* (heterogeneous nuclear ribonucleoprotein U-like 1)
	41788394(C/T)	Intron 7	*HNRNPUL1* (heterogeneous nuclear ribonucleoprotein U-like 1)
	47580255(A/G)	Intron 11	*ZC3H4* (zinc finger CCCH-type containing 4)
	42344914(G/A)	Intron 1	*LYPD4* (LY6/PLAUR domain containing 4)
	45623510(C/T)	Intron 1	*LRRC68* (leucine rich repeat containing 68)
	56531237(C/G)	Intron 5	*NLRP5* (NLR family, pyrin domain containing 5)
